# Electronic health record-wide association study for atrial fibrillation in a British cohort

**DOI:** 10.3389/fcvm.2023.1204892

**Published:** 2023-09-28

**Authors:** Sheng-Chia Chung, Amand F. Schmit, Gregory Y. H. Lip, Rui Providencia

**Affiliations:** ^1^ Institute of Health Informatics, University College London, London, United Kingdom; ^2^ Institute of Cardiovascular Science, University College London, London, United Kingdom; ^3^ Liverpool Centre for Cardiovascular Science at University of Liverpool, Liverpool John Moores University and Liverpool Heart & Chest Hospital, Liverpool, United Kingdom; ^4^ Danish Center for Health Services Research, Department of Clinical Medicine, Aalborg University, Aalborg, Denmark; ^5^ Barts Heart Centre, Barts Health NHS Trust, London, United Kingdom

**Keywords:** arrhythmia, electronic health records, clinical visits, population study, hospitalization, primary care

## Abstract

**Background:**

Atrial fibrillation (AF) confers a major healthcare burden from hospitalisations and AF-related complications, such as stroke and heart failure. We performed an electronic health records-wide association study to identify the most frequent reasons for healthcare utilization, pre and post new-onset AF.

**Methods:**

Prospective cohort study with the linked electronic health records of 5.6 million patients in the United Kingdom Clinical Practice Research Datalink (1998–2016). A cohort study with AF patients and their age-and sex matched controls was implemented to compare the top 100 reasons of frequent hospitalisation and primary consultation.

**Results:**

Of the 199,433 patients who developed AF, we found the most frequent healthcare interactions to be cardiac, cerebrovascular and peripheral-vascular conditions, both prior to AF diagnosis (41/100 conditions in secondary care, such as cerebral infarction and valve diseases; and 33/100 conditions in primary care), and subsequently (47/100 conditions hospital care and 48 conditions in primary care). There was a high representation of repeated visits for cancer and infection affecting multiple organ systems. We identified 10 novel conditions which have not yet been associated with AF: folic acid deficiency, pancytopenia, idiopathic thrombocytopenic purpura, seborrheic dermatitis, lymphoedema, angioedema, laryngopharyngeal reflux, rib fracture, haemorrhagic gastritis, inflammatory polyneuropathies.

**Conclusion:**

Our nationwide data provide knowledge and better understanding of the clinical needs of AF patients suggesting: (i) groups at higher risk of AF, where screening may be more cost-effective, and (ii) potential complications developing following new-onset AF that can be prevented through implementation of comprehensive integrated care management and more personalised, tailored treatment.

**Clinical trial registration:**

NCT04786366

## Background

Atrial fibrillation (AF) is the most frequently sustained cardiac arrhythmia worldwide (1). AF is a clinically heterogeneous condition that can have multiple distinct presentations, ranging from an absence of symptoms to palpitations, and from the development of heart failure or stroke to other cardiovascular and non-cardiovascular complications (2). Identification of disease associations or risk factors can be done following a hypothesis-driven approach based on the understanding of the disease pathophysiology, or through a “*hypothesis-free*” method, which may be advantageous in cases where there is an incomplete understanding of the pathophysiology, for example, genome-wide association studies (GWAS) to identify novel associations & involved pathways (3).

Similarly to GWAS, electronic health records (EHR) have been previously used for such an approach (4), and could be used for further characterizing the clinical heterogeneity of AF in the UK population through the identification of disease associations. This is important given that AF confers a major healthcare burden from hospitalisations and AF-related complications, such as stroke and heart failure.

We therefore conducted an EHR-wide association study to investigate the most frequent reasons for healthcare interactions pre- and post-AF diagnosis, as compared to individuals without AF, in both primary and secondary care (i.e., primary care consultations and hospitalizations). The findings of the study would provide a better understanding of additional healthcare utilization required in individuals prone to AF and may offer opportunities for screening and potentially preventing or delaying the development of the arrhythmia. Meanwhile, frequent clinical visits following an AF diagnosis help identify the progression of the disease and may be used to define AF clinical sub-phenotypes in routine care, whereas groups of AF patients with specific health service interaction patterns may benefit from tailored holistic or integrated care management approaches.

## Methods

The Clinical Practice Research Datalink (CPRD) was established in 1987 (5) and as of 2018 includes 7,998,501 patients in the UK with linked data of primary care consultation, hospital data (Hospital Episodes Statistics, HES), national cancer registry (National Cancer Intelligence Network) and death registry data (Office for National Statistics, ONS) (6, 7). The data are generally representative of the age, gender and geographic distribution of the UK population (5), and showed high quality and completeness of clinical information recorded (6–8). The present study was approved by the Medicines and Healthcare products Regulatory Agency Independent Scientific Advisory Committee [17_205].

Our cohort was composed of individuals aged 18 years or older registered in the current primary care practice for at least one year. The study period was between 1 January 1998 and 31 May 2016, and individuals were excluded if they had a prior history of AF before study entry. AF was defined from the International Classification of Diseases (ICD), tenth revision as I48 from HES and Read codes G573400, G573500, 3,272.00, G573000, G573300, G573.00, G573z00 from CPRD.

We implemented a matched case-control study for investigating the most common problems and comorbidities of AF patients when compared to controls. The primary diagnoses at their general practice (GP) consultation and hospitalisation within five years before and after the diagnosis date in individuals with AF were compared with that of their age and sex-matched controls. For each AF patient, the most frequent GP visits recorded in CPRD and primary diagnosis for hospital admissions documented in HES were identified within five years before and after the initial AF diagnosis. Similarly, we summarised the top conditions for most frequent clinical visits pre and post-index date in matched controls.

We reported the frequencies (%) of the conditions as the most frequent reasons for GP visits and hospital admissions in AF patients and their matched controls. We then summarised the differences between individuals with and without AF by relative frequency (frequency ratio) and reported the leading 100 conditions with descending frequency ratios. The uncertainty of the ratios was estimated with bootstrap distributions based on 2,000 samples with the Balanced Bootstrap Resampling method. We reported the clinical conditions requiring hospitalisation by disease groups (Table 1). We performed the analyses in Statistical Analysis System (version 9.4) and R (version 3.6.1).

**Table 1:** Leading 100 frequent causes for hospitalisation or general practise consultation prior to the index date comparing AF and matched controls.

Primary care		Secondary care	
Cardiac-Related (16)	*a. Arrhythmia*(*7*)*:* 6. Atrial flutter, 23. Cardiac arrhythmias, 29. Heart beats irregular, 45. Paroxysmal atrial tachycardia, 57. Sinus tachycardia, 76. Supraventricular tachycardia NOS, & 81. Ectopic beats. *b. Heart failure (1):* 30. Cardiac failure. c. Heart valve disease (4): 7. Aortic stenosis, 10. Mitral stenosis, 33. Aortic regurgitation alone, cause unspecified, & 53. Mitral regurgitation, *d. Cardiomyopathy (0):* *e. Ischaemic heart disease (3):* 4. Acute non-ST myocardial infarction, 5. Acute ST myocardial infarction, & 67. Double coronary vessel disease, *f. Other (1):* 59. Ventricular septal defect.	Cardiac-Related (22)	*a. Arrhythmia (9):* 9. Supraventricular tachycardia (I47.1), 14. Palpitations (R00.2), 18. Atrioventricular block, first degree (I44.0), 26. Sick sinus syndrome (I49.5), 40. Cardiac arrest with successful resuscitation (I46.0), 53. Trifascicular block (I45.3), 56. Other specified cardiac arrhythmias (I49.8), 60. Paroxysmal tachycardia, unspecified (I47.9), & 100. Bradycardia, unspecified (R00.1). *b. Heart failure (3):* 10. Heart failure, unspecified (I50.9), 50. Cardiomegaly (I51.7), & 55. Heart failure (I50.0/I50.1). *c. Heart valve disease (5):* 4. Disorders of both mitral and aortic valves (I08.0), 7. Mitral (valve) insufficiency (I34.0), 11. Aortic (valve) insufficiency (I35.1), 12. Aortic (valve) stenosis (I35.0), & 72. Mitral (valve) prolapse (I34.1). *d. Cardiomyopathy (1):* 37. Dilated cardiomyopathy (I42.0). *Ischaemic heart disease (3):* 25. Subsequent myocardial infarction of anterior wall (I22.0), 64. Subsequent myocardial infarction of unspecified site (I22.9), & 95. Acute subendocardial myocardial infarction (I21.4). *Other (1):*22. Atrial septal defect (Q21.1).
Cerebrovascular (7)	20. Left-sided CVA, 38. Stroke due to cerebral arterial occlusion, 44. CVA - cerebral artery occlusion, 77. Cerebral infarction NOS, 80. Cerebral infarction due to unspecified occlusion or stenosis of unspecified cerebral artery, 87. Right sided cerebral infarction, & 99. Cerebrovascular disease NOS.	Cerebrovascular (6)	5. Cerebral infarction due to unspecified occlusion or stenosis of cerebral arteries (I63.5), 32. Cerebral infarction due to thrombosis of cerebral arteries (I63.3), 36. Other cerebral infarction (I63.8), 42. Cerebral infarction due to unspecified occlusion or stenosis of precerebral arteries (I63.2), 87. Stroke, not specified (I64/I63.9), & 96. Multi-infarct dementia (F01.1).
Peripheral or other vascular (9)	3. Primary pulmonary hypertension, 13. Mixed venous and arterial leg ulcer, 17. Venous ulcer of leg, 47. Arterial leg ulcer, 64. Peripheral oedema, 84. Lipodermatosclerosis, 89. Venous ulcer of leg, 95. Raised blood pressure reading, & 98. AAA - Abdominal aortic aneurysm without mention of rupture.	Peripheral or other vascular (11)	15. Hypertensive renal disease with renal failure (I12.0), 23. Thoracic aortic aneurysm, without mention of rupture (I71.2), 30. Atherosclerosis of arteries of extremities (I70.2), 39. Embolism and thrombosis of arteries of upper extremities (I74.2), 62. Orthostatic hypotension (I95.1), 66. Lymphoedema, not elsewhere classified (I89.0), 71. Disease of pericardium, unspecified (I31.9), 75. Varicose veins of lower extremities with inflammation (I83.1), 81. Abdominal aortic aneurysm, ruptured (I71.3), 90. Disorder of arteries and arterioles, unspecified (I77.9), & 91. Varicose veins of lower extremities with both ulcer (I83.2).
Bleeding/Haemorrhagic complications (1)	91. Intracerebral haemorrhage.	Bleeding/Haemorrhagic complications (2)	24. Subdural haemorrhage (acute)(nontraumatic) (I62.0), & 89. Vitreous haemorrhage (H43.1).
Infection (13)	15. Acute lower respiratory tract infection, 22. Acute infective otitis externa, 27. Cellulitis NOS, 35. Paronychia of toe, 42. Sepsis, 48. Infected dermatitis, 58. Recurrent chest infection, 63. Other cellulitis and abscess, 66. Infection toe, 68. Nasal vestibulitis, 71. Acute upper respiratory tract infection, 72. Infective otitis externa, & 85. Pilonidal sinus/cyst.	Infection (4)	19. Acute and subacute infective endocarditis (I33.0), 27. Sepsis, unspecified (A41.9), 41. Other specified bacterial intestinal infections (A04.8), & 68. Viral intestinal infection, unspecified (A08.4).
Cancer (4)	21. Malignant neoplasm of oesophagus, 26. Oesophageal cancer, 41. Malignant neoplasm of trachea, bronchus and lung, 97. Polycythaemia vera,	Cancer (22)	1. Malignant neoplasm: Upper lobe, bronchus or lung (C34.1), 2. Malignant neoplasm: Lower lobe, bronchus or lung (C34.3), 3. Malignant neoplasm: Bronchus or lung, unspecified (C34.9), 6. Malignant neoplasm of ovary (C56), 8. Malignant neoplasm: Oesophagus, unspecified (C15.9), 13. Malignant neoplasm: Cardia (C16.0), 16. Diffuse large B-cell lymphoma (C83.3), 28. Non-Hodgkin lymphoma, unspecified (C85.9), 31. Chronic lymphocytic leukaemia of B-cell type (C91.1), 38. Mesothelioma of pleura (C45.0), 45. B-cell lymphoma, unspecified (C85.1), 48. Polycythaemia vera (D45), 52. Malignant neoplasm: Hepatic flexure (C18.3), 57. Malignant neoplasm: Malignant melanoma of upper limb (C43.6), 58. Secondary malignant neoplasm of lung (C78.0), 59. Malignant neoplasm without specification of site (C80), 65. Malignant neoplasm: Splenic flexure (C18.5), 70. Other non-follicular lymphoma (C83.8), 80. Malignant neoplasm: Upper-outer quadrant of breast (C50.4), 82. Multiple myeloma (C90.0), 97. Malignant neoplasm: Head of pancreas (C25.0), & 98. Intraductal carcinoma *in situ* (D05.1).
Respiratory conditions (6)	1. COPD, 31. Acute exacerbation of chronic obstructive airways disease, 60. Acute exacerbation of asthma, 73. Moderate chronic obstructive pulmonary disease, 74. Severe chronic obstructive pulmonary disease, 78. Diffuse pulmonary fibrosis,	Respiratory conditions (6)	21. Emphysema, unspecified (J43.9), 51. Pneumothorax, unspecified (J93.9), 61. Pleural effusion, not elsewhere classified (J90), 77. Pneumonitis due to food and vomit (J69.0), 83. Chronic obstructive pulmonary disease with acute lower respiratory infection (J44.0), & 92. Pulmonary collapse (J98.1).
Endocrine, nutritional or metabolic (10)	12. Folic acid deficiency, 16. Impaired fasting glycemia, 18. Impaired glucose tolerance, 19. Insulin-dependent diabetes mellitus, 28. Impaired glucose tolerance, 32. Hyponatraemia, 49. Iron deficiency, 62. Non-proliferative diabetic retinopathy, 83. Background diabetic retinopathy, & 100. Glucose intolerance.	Endocrine, nutritional or metabolic (6)	29. Insulin-dependent diabetes mellitus with peripheral circulation complications (E10.5), 35. Disorders of calcium metabolism (E83.5), 74. Hyperkalaemia (E87.5), 76. Non-insulin-dependent diabetes mellitus with peripheral circulatory complications (E11.5), 84. Hypo-osmolality and hyponatraemia (E87.1), & 99. Non-insulin-dependent diabetes mellitus with ketoacidosis (E11.1).
Gastrointestinal (3)	55. Constipation NOS, 69. Small bowel obstruction NOS, & 79. Alcoholic cirrhosis of liver.	Gastrointestinal (7)	20. Polyp of stomach and duodenum (K31.7), 33. Acute haemorrhagic gastritis (K29.0), 46. Alcoholic cirrhosis of liver (K70.3), 69. Obstruction of bile duct (K83.1), 78. Perforation of intestine (nontraumatic) (K63.1), 85. Oesophageal varices without bleeding (I85.9), & 94. Diverticular disease of large intestine with perforation and abscess (K57.2),
Renal (1)	56. Proteinuria.	Renal (3)	17. Chronic kidney disease (N18.0), 47. Acute renal failure, unspecified (N17.9), & 93. Other chronic renal failure (N18.8).
Haematological (3)	40. Chronic anaemia, 43. Microcytic hypochromic anaemia, & 50. Pancytopenia NOS.	Haematological (2)	49. Thrombocytopenia, unspecified (D69.6), & 88. Idiopathic thrombocytopenic purpura (D69.3).
Osteoarticular & muscular disorders (5)	9. Fragility fracture, 24. Arthralgia of hip, 25. Arthropathy NOS, 37. Leg pain, & 51. Polyarthropathy NEC.	Osteoarticular & muscular disorders (4)	54. Localized swelling, mass and lump, lower limb (R22.4), 67. Fracture of rib (S22.3), 73. Lumbago with sciatica (M54.4), & 86. Synovial cyst of popliteal space [Baker] (M71.2).
Frailty or Multimorbidity (9)	2. Uncertain Diagnosis, 11. Tired all the time, 34. Catheter complications, 36. Geriatric fall, 39. Ulcer of skin, 70. Slurred speech, 75. Confusion, 92. Wet senile macular degeneration, & 96. Chronic skin ulcer.	Frailty or Multimorbidity (2)	44. Malaise and fatigue (R53), & 79. Decubitus ulcer and pressure area (L89).
Other (13)	8. Pruritus vulvae, 14. Seborrhoeic dermatitis capitis, 46. Moderate depressive episode, 52. Scalp psoriasis, 54. Breathlessness, 61. Bullous pemphigoid, 65. Alcohol withdrawal syndrome, 82. Homonymous hemianopia, 83. Injury and poisoning NOS, 86. Problem with vaginal pessary, 88. Generalized anxiety disorder, 90. Skin lesion, & 94. Ingrowing great toe nail.	Other (3)	34. Dyspnoea (R06.0), 43. Delirium, unspecified (F05.9), & 63. Precordial pain (R07.2).

## Results

Between 1 January 1998 and 31 May 2016, 7,998,501 participants in CPRD were eligible for linkage. After excluding individuals aged <18 at study entry (*n* = 1,061,689), not registered with primary care during the study period (*n* = 407,430), with invalid entry and exit dates into the study (*n* = 927,579) and with a previous diagnosis of AF (*n* = 44,238) we obtained a study cohort of 5,557,405 individuals. Over a median of 10.3 years of follow-up (interquartile range: 4.8–15.0) there were 199,433 (3.6%) patients with a diagnosis of new-onset AF, who constituted our cases. These were matched to an equal number of age and sex-matched non-AF controls. The mean age was 75.8 years (SD 12.7) in patients and 75.7 years (SD 12.7) in matched controls.

We observed a predominance of repeated hospitalisation or GP consultation owing to cardiac, cerebrovascular and peripheral vascular conditions prior to AF diagnosis (33 of the top 100 causes in primary care and 41 of the top 100 causes in secondary care) and also subsequently (47 of the top 100 causes in primary care and 48 of the top 100 causes in secondary care). A synthesis of our findings by disease groups is presented in Tables 1, 2.

**Table 2:** Leading 100 frequent causes for hospitalisation or general practise consultation following the index date comparing AF and matched controls.

Primary care		Secondary care	
Cardiac-Related (29)	*a. Arrhythmia (10):* 4. Paroxysmal atrial tachycardia, 5. Cardiac dysrhythmia NOS, 19. Cardiac arrhythmias, 25. Tachycardia (unspecified), 29. Cardiac dysrhythmias, 40. Palpitations, 46. Heart beats irregular, 53. Ventricular tachycardia, 87. Cardiac arrest, & 95. Supraventricular tachycardia NOS. *b. Heart failure (4):* 42. Pulmonary oedema NOS, 44. Left ventricular systolic dysfunction, 52. Cardiomegaly, & 79. Heart failure. *c. Heart valve disease (4):* 15. Mitral regurgitation, 31. Mitral stenosis, 66. Aortic stenosis alone, cause unspecified, & 96. Aortic regurgitation alone, cause unspecified. *d. Cardiomyopathy (2):* 1. Cardiomyopathy, & 24. Ischaemic cardiomyopathy. *e. Ischaemic heart disease (7):* 16. Acute non-ST segment elevation myocardial infarction, 20. Acute ST-elevation myocardial infarction, 22. Coronary artery bypass occlusion, 34. Acute coronary syndrome, 41. Triple vessel disease of the heart, 81. Single coronary vessel disease, & 92. Coronary artery disease. *f. Other (2):* 21. Echocardiogram abnormal, & 45. Acute pericarditis.	Cardiac-Related (28)	*a. Arrhythmia (7):* 18. Ventricular fibrillation and flutter (I49.0), 34. Ventricular tachycardia (I47.2), 39. Supraventricular tachycardia (I47.1), 57. Cardiac arrest with successful resuscitation (I46.0), 81. Sick sinus syndrome (I49.5), 93. Other and unspecified atrioventricular block (I44.3), 100. Tachycardia, unspecified (R00.0). *b. Heart failure (2)*: 8. Fluid overload (E87.7), & 98. Heart failure (I50.0/I50.1). *c. Heart valve disease (8):* 2. Disorders of valves (I08.1), 3. Combined disorders of valves (I08.3), 5. Mitral (valve) insufficiency (I34.0), 11. Disorders of both mitral and aortic valves (I08.0), 28. Mechanical complication of heart valve prosthesis (T82.0), 35. Aortic (valve) stenosis with insufficiency (I35.2), 54. Aortic (valve) insufficiency (I35.1), & 62. Aortic (valve) stenosis (I35.0), *d. Cardiomyopathy (5):* 4. Dilated cardiomyopathy (I42.0), 20. Cardiomyopathy, unspecified (I42.9), 31. Obstructive hypertrophic cardiomyopathy (I42.1), 51. Other hypertrophic cardiomyopathy (I42.2), & 70. Ischaemic cardiomyopathy (I25.5). *e. Ischaemic heart disease (2):* 56. Other forms of acute ischaemic heart disease (I24.8), & 82. Acute ischaemic heart disease, unspecified (I24.9). *f. Other (4):* 6. Atrial septal defect (Q21.1), 19. Poisoning: Cardiac-stimulant glycosides and drugs of similar action (T46.0), 26. Acute pericarditis, unspecified (I30.9), & 66. Disease of pericardium, unspecified (I31.9).
Cerebrovascular (7)	10. Right-sided cerebral infarction, 18. Infarction of basal ganglia, 27. Stroke due to cerebral arterial occlusion, 39. Cerebral infarction due to thrombosis of cerebral artery, 48. Cerebellar stroke syndrome, 65. Cerebral infarction due to unspecified occlusion or stenosis of unspecified cerebral artery, & 82. Stroke unspecified.	Cerebrovascular (8)	1. Cerebral infarction (I63.4), 7. Anoxic brain damage, not elsewhere classified (G93.1), 22. Cerebral infarction due to thrombosis of cerebral artery (I63.3), 29. Cerebral infarction due to unspecified occlusion or stenosis of cerebral arteries (I63.5), 33. Cerebral atherosclerosis (I67.2), 44. Cerebral infarction due to embolism of precerebral arteries (I63.1), 52. Cerebral infarction due to thrombosis of precerebral arteries (I63.0), & 53. Cerebral infarction due to unspecified occlusion or stenosis of precerebral arteries (I63.2),
Peripheral or other vascular (10)	7. primary pulmonary hypertension, 9. Diabetes mellitus with peripheral circulatory disorder, 54. Bowel infarction, 62. Low blood pressure reading, 67. Mixed venous and arterial leg ulcer, 70. Abdominal aneurysm which has ruptured, 76. Pulmonary embolism, 83. Ischaemia of legs, 85. Acute cor pulmonale, 91. Arterial leg ulcer.	Peripheral or other vascular (4)	9. Thoracic aortic aneurysm (I71.2), 30. Primary pulmonary hypertension (I27.0), 76. Other specified complications of cardiac and vascular prosthetic devices, implants and grafts (T82.8), & 95. Vascular disorder of intestine, unspecified (K55.9).
Bleeding/Haemorrhagic complications (1)	13. Subdural haemorrhage NOS.	Bleeding/Haemorrhagic complications (7)	23. Haemorrhagic disorder due to circulating anticoagulants (D68.3), 49. Conjunctival haemorrhage (H11.3), 69. Haemorrhage and haematoma complicating a procedure (T81.0), 77. Traumatic subarachnoid haemorrhage (S06.6), 79. Intracerebral haemorrhage in hemisphere, cortical (I61.1), 80. Haemarthrosis (M25.0), & 89. Traumatic haemopneumothorax (S27.2).
Infection (11)	2. Conjunctivitis, 11. Empyema, 35. Clostridium difficile infection, 36. Sepsis, 43. Viral gastroenteritis, 56. Chronic osteomyelitis, 59. Other aspiration pneumonia as a complication of care, 60. Other specified pneumonia or influenza, 84. Peritonitis, 89. Pneumonia & influenza, 97. Basal pneumonia due to unspecified organism.	Infection (19)	10. Gastroenteritis and colitis of unspecified origin (A09.9), 15. Acute pancreatitis, unspecified (K85.9), 21. Pneumonia due to other streptococci (J15.4), 25. Candidiasis of other sites (B37.8), 27. Acute gastroenteropathy due to Norwalk agent (A08.1), 36. Acute and subacute infective endocarditis (I33.0), 37. Other and unspecified gastroenteritis and colitis of infectious origin (A09.0), 38. Pyonephrosis (N13.6), 41. Legionnaires’ disease (A48.1), 45. Pneumonia due to Haemophilus influenzae (J14), 48. Osteomyelitis, unspecified (M86.9), 59. Endocarditis, valve unspecified (I38), 61. Calculus of bile duct with cholecystitis (K80.4), 63. Streptococcal infection, unspecified site (A49.1), 71. Bacterial pneumonia, unspecified (J15.9), 72. Acute appendicitis, other and unspecified (K35.8), 83. Unspecified viral encephalitis (A86), 87. Infection of intervertebral disc (pyogenic) (M46.3), & 96. Other streptococcal sepsis (A40.8),
Cancer (8)	28. Acute myeloid leukaemia, 32. Hepatocelullar carcinoma, 51. Glioblastoma multiforme, 55. Chronic myeloid leukaemia, 58. Malignant neoplasm of oesophagus NOS, 75. Meningiomas, 80. Malignant pleural effusion, 99. Malignant Neoplasm of endometrium of corpus uteri	Cancer (4)	32. Peripheral T-cell lymphoma, not elsewhere classified (C84.4), 84. Malignant neoplasm: Floor of mouth, unspecified (C04.9), 85. Other specified types of non-Hodgkin lymphoma (C85.7), & 97. Hodgkin lymphoma, unspecified (C81.9).
Respiratory conditions (8)	26. Respiratory failure, 33. Obstructive sleep apnoea, 61. Respiratory failure, 68. Pleural effusion NOS, 73. Laryngopharyngeal reflux, 86. Pleural effusion NOS, 90. Aspiration pneumonitis, 93. Acute exacerbation of chronic obstructive airways disease.	Respiratory conditions (2)	47. Chronic respiratory failure (J96.1), & 64. Tracheostomy malfunction (J95.0).
Endocrine, nutritional or metabolic (7)	6. Vitamin D deficiency, 23. Hypomagnesemia, 30. Chronic pancreatitis, 47. Insulin-dependent diabetes mellitus, 64. Prediabetes, 78. Thyrotoxicosis, & 88. Folic acid deficiency.	Endocrine, nutritional or metabolic (5)	16. Other obesity (E66.8), 24. Thyrotoxicosis, unspecified (E05.9), 42. Thyrotoxicosis with toxic multinodular goitre (E05.2), 60. Hypokalaemia (E87.6), & 94. Other chronic pancreatitis (K86.1).
Gastrointestinal (3)	8. Acid reflux, 49. Perforation of intestine & 57. Cirrhosis and chronic liver disease.	Gastrointestinal (6)	17. Fistula of intestine (K63.2), 65. Postoperative intestinal obstruction (K91.3), 68. Alcoholic hepatitis (K70.1), 75. Alcoholic cirrhosis of liver (K70.3), 78. Other specified noninfective gastroenteritis and colitis (K52.8), & 92. Barrett's oesophagus (K22.7).
Renal (2)	63. Acute renal failure, 69. Impaired renal function.	Renal (4)	13. Chronic kidney disease stage 5 (N18.5), 46. Acute renal failure with tubular necrosis (N17.0), 88. Hydronephrosis with ureteral stricture, not elsewhere classified (N13.1), & 99. Acute renal failure, unspecified (N17.9).
Haematological (2)	72. Idiopathic thrombocytopenic purpura, & 74. Pancytopenia acquired.	Haematological (2)	40. Thrombocytopenia, unspecified (D69.6), & 56. Anaemia in other chronic diseases classified elsewhere (D63.8).
Osteoarticular & muscular disorders (3)	14. Fragility fracture, 38. Callosity on foot, & 50. Pseudogout.	Osteoarticular & muscular disorders (3)	73. Superficial injury of shoulder and upper arm, unspecified (S40.9), 74. Traumatic ischaemia of muscle (T79.6), & 90. Fracture of sacrum (S32.1).
Frailty or Multimorbidity (4)	3. Multiple organ failure, 12. Mechanical complication of urethral catheter, 17. Symptoms, signs and ill-defined conditions, & 77. Wet senile macular degeneration.	Frailty or Multimorbidity (2)	12. Tendency to fall, not elsewhere classified (R29.6), & 55. Stage IV decubitus ulcer (L89.3).
Other (5)	37. Alcohol withdrawal syndrome, 71. Drug hypersensitivity NOS, 94. Incisional hernia, 98. Keloid scar, & 100. Angioedema.	Other (6)	14. Mental and behavioural disorders due to use of alcohol (F10.3), 43. Localization-related (focal) (partial) symptomatic epilepsy and epileptic syndromes with simple partial seizures (G40.1), 50. Visual disturbance, unspecified (H53.9), 67. Zoster ocular disease (B02.3), 86. Other inflammatory polyneuropathies (G61.8), & 91. Precordial pain (R07.2).

Compared to matched controls, the leading *cardiovascular* reasons of excess hospitalisation among individuals with AF prior to the initial AF diagnosis were “disorders of both mitral and aortic valves (I08.0)” (frequency ratio: 10.7), “cerebral infarction due to unspecified occlusion or stenosis of cerebral arteries (I63.5)” (frequency ratio: 10.6) and “mitral (valve) insufficiency (I34.0)” (frequency ratio: 9.2) (Supplementary Table S2). In the period prior to an AF diagnosis, the leading *non-cardiovascular* causes of more frequent hospitalisation compared AF to controls were “malignant neoplasm: Upper lobe, bronchus or lung (C34.1)”, “malignant neoplasm: Lower lobe, bronchus or lung (C34.3)”, and “malignant neoplasm: Bronchus or lung, unspecified (C34.9)” (frequency ratios: 19.0, 12.7, 12.7, respectively, Supplementary Table S2). Following an AF diagnosis, patients were more frequently hospitalised than controls for cardiovascular conditions such as “Cerebral infarction due to embolism of cerebral arteries (I63.4)”, “disorders of both mitral and tricuspid valves (I08.1)” and “combined disorders of mitral, aortic and tricuspid valves (I08.3)” (frequency ratio: 45.2, 30.6, 21.4, respectively) (Supplementary Table S3).

For frequent GP consultations, compared with matched controls, chronic obstructive pulmonary disease, primary pulmonary hypertension, and acute non-ST myocardial infarction were the leading conditions among AF patients prior to diagnosis. (Frequency ratio: 76.1, 35.1, 28.9, respectively. Supplementary Table S4) Individuals with AF were also more likely to frequently visit GP for conditions that seemed unclear at the time (uncertain diagnosis, frequency ratio: 48). After diagnosis, cardiomyopathy, conjunctivitis and multiple organ failure were the leading reasons for frequent GP consultations among AF patients vs. matched controls. (Supplementary Table S5).

**Figure 1:**
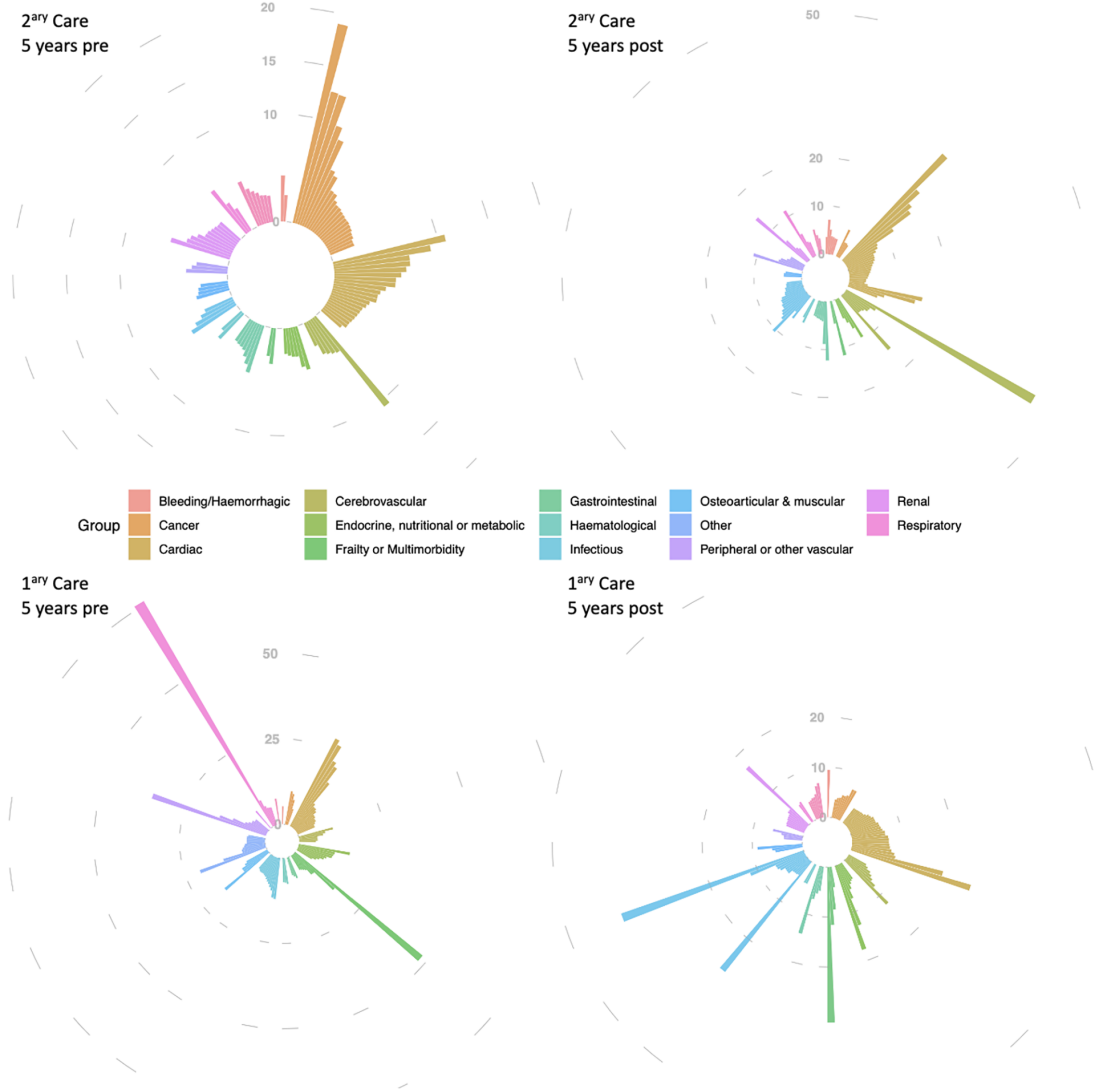
Top 100 reasons for hospitalisation (2ary care) and GP consultations (1ary care) in atrial fibrillation patients, compared to controls, within 5 years before and in the 5 years after new-onset AF diagnosis. The four iris-plots represent the top reasons (displayed as relative risk vs. controls) for healthcare utilization (primary and secondary care) prior and post new-onset AF. The 14 diagnostic groups are organized by different colours (legend) and clockwise as follows, as per the upper-left iris-plot: bleeding/haemorrhagic (12 h), cancer (1 h to 2 h), cardiac (3–4 h), cerebrovascular (5 h), “endocrine, nutritional or metabolic” & “frailty or multimorbidity” (6 h), gastrointestinal (7 h), haematological and infectious (8 h), osteoarticular & other (9 h), “peripheral other vascular” (10 h), renal (11 h), and respiratory (11–12 h). Different scales were used in the 4 iris-plots to accommodate with the different range of relative risks.

The four iris-plots in Figure 1 represent the top reasons (displayed as relative risk vs. controls, the length of each bar, based on Supplementary Tables S2–S5) for healthcare utilization (primary and secondary care) prior and post new-onset AF. The 14 diagnostic groups are organized by different colours and clockwise. The left upper-panel shows an over-representation of cardiac and cancer-related hospitalizations prior to new-onset AF and the right upper-panel shows over-representation of cardiac and cerebrovascular hospitalizations following new-onset AF. In primary care we observed is over-representation of cardiac-related visits both pre and post new-onset AF. Also, representation of consultations due to infectious disease diagnoses seems to increase following new-onset AF.

## Discussion

We report, using a hypothesis-free approach, the most frequent reasons for repeated hospitalisations or primary care consultations by comparing patients with AF and their non-AF-matched peers. We found a predominance of repeated clinical visits due to cardiac, cerebrovascular and peripheral vascular conditions in AF patients vs. matched controls, suggesting the development of arrhythmia may contribute to the aggravation or worsening cardiovascular or cerebrovascular health.

Our findings are in agreement with previous literature providing evidence on important AF complications and comorbidities, such as stroke (9), vascular dementia (10), valvular heart disease (11), myocardial infarction (12), hypertrophic cardiomyopathy (13), and heart failure (14), but also raise hypotheses towards some other unknown or less reported associations with other vascular disorders, such as peripheral artery disease, aortic aneurysms, which may require validation in a different cohort. Some of these associations, like peripheral artery disease (15), aortic aneurysms (16), and ventricular arrhythmias (17) have been suggested in the literature. Also, we have observed other less frequent causes that have been episodically reported (summarised in Supplementary Table S1).

Our data show excess repeated clinical visits affecting multiple organ systems, including for cancer (especially prior to an AF diagnosis) and frequent visits due to infection (mainly after new-onset AF). The association between AF and cancer had previously been reported (18, 19). Similarly, sepsis (20) and other forms of infection (21) have been reported as important complications associated with new-onset AF.

Our findings further reinforce the importance and high prevalence of comorbidities such as obesity (22) and associated conditions like obstructive sleep apnoea (23), and dysglycaemia in the AF population (24). Some of the reasons for repeated clinical visits occurring prior to an AF diagnosis are potentially reversible, with folic acid or vitamin D deficiency, hyponatraemia, hypomagnesemia, hyperkalaemia, iron deficiency, impaired fasting glycemia and glucose tolerance, being a few examples. Other reasons can have their onset delayed with proper intervention, such as type 2 diabetes mellitus (25) and hypertension. Our results suggest the timely detection and treatment or prevention of some of these conditions may potentially prevent or defer the development of AF. For example, the reduction of AF progression with weight loss has been previously demonstrated in the REVERSE-AF study (26).

Some of the reasons for clinical visits we observed may be the consequence of AF-related interventions. Following the diagnosis of AF, we observed an increase in repeated clinical visits for bleeding events, potentially resulting from the increase in the uptake of anticoagulants. Similarly, we also observe visits due to thyrotoxicosis after the diagnosis of AF. The association of hyperthyroidism with AF is well known (27), and with the frequent use of amiodarone in our cohort (28), thyrotoxicosis could partly be due to its concomitant use.

To the best of our knowledge, some of the reasons for frequent clinical visits experienced by AF patients in this study (folic acid deficiency, pancytopenia, idiopathic thrombocytopenic purpura, seborrheic dermatitis, lymphoedema, angioedema, laryngopharyngeal reflux, rib fracture, haemorrhagic gastritis and inflammatory polyneuropathies) have not yet been reported in the literature, and further studies would be recommended.

We reported for the first time the four iris-plots of healthcare utilization for AF patients. Each of these four iris-plots together provides an “*iris pattern*” similar to the one observed in the human eye. The “*iris pattern*” is considered unique to each individual, utilized as a biometric measure used for authentication, and known to more accurate than a fingerprint (29).

### Practice implications and future research

The current practice and management of AF emphasise more comprehensive management, as highlighted in the ABC (Atrial fibrillation Better Care) pathway (30) that promotes a holistic or integrated care approach that has been associated with improved clinical outcomes (31, 32) leading to its recommendations in guidelines (33, 34). Also, greater focus on comorbidities, such as the recent HEAD-2-TOES (35), going beyond the arrhythmia to associated cardiovascular and non-cardiovascular health conditions. We utilised nationwide data to provide knowledge and a better understanding of how AF patients frequently interact with the health system. Insights from healthcare contacts may facilitate improvements in preventive and treatment strategies for better management and prevention of subsequent outcomes. The shifting patterns of reasons for excess clinical visits in AF patients may contribute to the elucidation of the clinical heterogeneity of AF. Grouping the reasons AF patients seek medical attention by organ system or disease type, we propose the following AF clinical sub-phenotypes: (1) Vascular – associated with atherosclerotic, cerebrovascular and peripheral artery disease; (2) Myopathic – associated with heart failure, cardiomyopathies; (3) Valvular – associated with heart valve involvement; (4) Neoplastic – associated with cancer, potentially as part of inflammation or paraneoplastic syndrome; (5) Infectious – arising as a result of infection and in patients more prone to develop infection; (6) Endocrine or Metabolic – associated with obesity and impaired glucose tolerance or diabetes; (7) Senile – occurring as a result of aging and associated decline and comorbidities. Due to their nature and associated comorbidities, the partial overlap is likely and multiple clinical sub-phenotypes and treatment needs can coexist in the same individual. As an example, one patient may have AF with both Vascular and Metabolic clinical sub-phenotype, whilst a different patient can present with an overlap of Infectious and Senile AF clinical sub-phenotypes. Further studies are required for understanding the risk factors and prognosis of the proposed AF clinical phenotypes, and methods like cluster analyses or equivalent may be applicable. An in-depth understanding of these clinical phenotypes could not only improve the knowledge of the pathways involved and their interplay but optimise AF prevention and treatment.

A better understanding of the conditions and causes for frequent clinical visits preceding the diagnosis of AF may help identify higher-risk subgroups of patients, such as patients with cancer, cardiovascular, cerebrovascular, peripheral vascular disease, respiratory or gastrointestinal disease, for whom a targeted screening strategy may derive more pronounced benefits, including savings in healthcare costs (36). This is important given systematic screening of the general or certain population subgroups has yielded low or negative net benefits (37).

### Research implications

An electronic health record (EHR)-wide association study has been previously conducted for better characterizing COVID-19 outcomes (4) but has not been utilized in the field of cardiovascular disease. In this first AF EHR-wide association study, we found the excess clinical visits associated with AF differed by the primary and secondary care and by before and after the diagnosis date. Our experience indicates the importance of considering the type of healthcare and timing of individuals interacting with the care system in applying the EHR-wide association study method.

In this paper we present the “*iris pattern*” for AF as a disease interacting with primary and secondary care. Even though “*iris patterns*” have not yet been reported for other diseases, we can hypothesize that as risk factors and their combination differ across different diseases, different “*iris patterns*” of healthcare utilization, possibly unique to each disease entity, may also exist. This hypothesis requires further clarification, but if proven may help tackle disease specific aspects at primary and secondary prevention level (i.e., prior and post disease onset).

### Strength and limitations

The strengths of our research are the power of electronic health records enabling high-resolution analyses for detailed clinical conditions. The generalisability of our findings based on population electronic health records from routine care allows the findings to be applicable to the general clinical practice in comparable populations. The comprehensive scope of conditions that occur in the AF population and their non-AF peers as documented in our analyses raises the possibility and need for a more individualized system contemplating the needs of specific patients or groups of patients (AF clinical sub-phenotypes), some of whom represent clinically complex patients (38). However, there were also some limitations, including the absence of complete information on AF types (paroxysmal, persistent, permanent). However, the clinical utility of such a temporal pattern/episode-based classification remains inconclusive (39). Additionally, even though CPRD patients are thought to be broadly representative of the UK general population in terms of age, sex and ethnicity (40), and a recent study suggesting that CPRD populations may be representative of the general UK populations in terms of socioeconomic status and rural-urban classification (41), we lack the evidence to say if this population is truly representative of the total AF population in the UK. An ongoing study to develop an artificial intelligence model to identify AF patients in the UK may provide an answer to this important area of uncertainty (42). The study will compare two CPRD datasets: CPRD-Gold (which we have utilized to address our research protocol aiming at clarifying the natural history of AF in the UK) and CPRD-Aurum (established in 2017 and comprising 26.9 million additional individuals). Finally, our analyses did not utilize a competing risk framework and have not accommodated for this potential source of bias. Mortality is known to be higher in AF patients when compared to peers (12). Therefore, it is expected that mortality will be a competitive event in relation to the evaluated diagnoses in the post-AF comparisons performed in our analyses. The earlier mortality of AF patients may have precluded the occurrence of the events of interest being assessed, potentially leading to sub-diagnosis.

## Conclusion

In this EHR-wide study for AF, we found cardiac, cerebrovascular and other vascular problems are still amongst the most frequent comorbidities in this population, but other disease groups, such as infection and cancer were also frequent. The need for early detection of AF and management of comorbidities may inform targeted early diagnosis and optimal care strategies for AF.

## Data Availability

The data analyzed in this study is subject to the following licenses/restrictions: Data may be obtained from a third party and are not publicly available. Data used in this study were accessed through NHS Digital that is subject to protocol approval and cannot directly be shared. Requests to access these datasets should be directed to Medicines Healthcare products Regulatory Agency - Clinical Practice Research Datalink, https://cprd.com.
