# Supplementary material for: Electronic health record-wide association study for atrial fibrillation in a British cohort
Source: Front Cardiovasc Med. 2023 Sep 28;10:1204892. doi: 10.3389/fcvm.2023.1204892 (PMC10569421; doi:10.3389/fcvm.2023.1204892)

Supplementary appendix of the study

Electronic health record-wide association study comparing individuals with and without atrial fibrillation in the United Kingdom

Sheng-Chia Chung, A. Floriaan Schmit, Gregory Lip, Rui Providencia

Table of content:

Supplementary Table S1: External validation for some of the less well-known identified AF associations

Supplementary Table S2: Top 100 reasons for hospitalisation in atrial fibrillation patients, compared to controls, within 5 years pre incident AF diagnosis.

Supplementary Table S3: Top 100 reasons for hospitalisation in atrial fibrillation patients, compared to controls, in the 5 years post incident AF diagnosis.

Supplementary Table S4: Top 100 reasons for GP consultations in atrial fibrillation patients, compared to controls, within 5 years pre incident AF diagnosis.

Supplementary Table S5: Top 100 reasons for GP consultations in atrial fibrillation patients, compared to controls, in the 5 years post incident AF diagnosis.

Supplementary Table S1: External validation for some of the less well-known identified AF associations

| **Association** | **Reference** | **Cohort / Dataset** |
| --- | --- | --- |
| Anaemia and Low Iron levels | Lim et al. 2020 | Korean National Health Insurance Service database |
| Anxiety | Eaker et al. 2005 | Framingham Heart Study |
| Asthma | Cepelis et al. 2018 | The Trøndelag Health Study (HUNT) |
| Depression | Kim et al. 2022 | Korean National Health Insurance Service database |
| Epilepsy | Doege et al. 2022 | IQVIA Disease Analyzer database in Germany |
| Fragility fractures | Sherer et al. 2020 | Framingham Heart Study |
| Gastroesophageal reflux disease | Maret-Ouda et al. 2022 | Swedish Nationwide adult residents |
| Hypokalemia | Krijthe et al. 2013 | The Rotterdam Study |
| Hypomagnesemia | Khan et al. 2013 | Framingham Heart Study |
| Proteinuria | Molnar et al. 2017 | Administrative healthcare databases in Ontario |
| Type-1 Diabetes mellitus | Dahlqvist et al. 2017 | Swedish National Diabetes Registry & Swedish Population Register |
| Varicose veins | Hu et al. 2022 | Taiwan National Health Insurance Research Database |
| Vitamin D deficiency | Rahimi et al. 2021 | Meta-analysis of 12 observational studies |
| Zoster | Cha et al. 2018 | Korean National Health Insurance Service database |

References – Supplementary Material

1. Lim WH, Choi EK, Han KD, Lee SR, Cha MJ, Oh S. Impact of Hemoglobin Levels and Their Dynamic Changes on the Risk of Atrial Fibrillation: A Nationwide Population-Based Study. Sci Rep. 2020;10:6762.
2. Eaker ED, Sullivan LM, Kelly-Hayes M, D’Agostino RB, Benjamin EJ. Tension and anxiety and the prediction of the 10-year incidence of coronary heart disease, atrial fibrillation, and total mortality: the Framingham offspring study. *Psychosomatic Medicine.*2005;**67**:692–696.
3. Cepelis A, Brumpton BM, Malmo V, Laugsand LE, Loennechen JP, Ellekjær H, Langhammer A, Janszky I, Strand LB. Associations of Asthma and Asthma Control With Atrial Fibrillation Risk: Results From the Nord-Trøndelag Health Study (HUNT). JAMA Cardiol. 2018;3:721-728.
4. Kim YG, Lee KN, Han KD, Han KM, Min K, Choi HY, Choi YY, Shim J, Choi JI, Kim YH. Association of Depression With Atrial Fibrillation in South Korean Adults. JAMA Netw Open. 2022;5:e2141772.
5. Doege C, Luedde M, Kostev K. Atrial fibrillation is associated with a subsequent epilepsy diagnosis independent of stroke: A retrospective matched administrative cohort study on 149,632 patients. Epilepsy Behav. 2022;132:108721.
6. Sherer JA, Huang Q, Kiel DP, Benjamin EJ, Trinquart L. Atrial Fibrillation and the Risk of Subsequent Fracture. Am J Med. 2020;133:954-960.
7. Maret-Ouda J, Santoni G, Xie S, Rosengren A, Lagergren J. Objectively confirmed gastroesophageal reflux disease and risk of atrial fibrillation: a population-based cohort study in Sweden.Eur J Gastroenterol Hepatol. 2022 Nov 1;34(11):1116-1120.
8. Krijthe BP, Heeringa J, Kors JA, Hofman A, Franco OH, Witteman JC, Stricker BH. Serum potassium levels and the risk of atrial fibrillation: the Rotterdam Study. Int J Cardiol. 2013;168:5411-5.
9. Khan AM, Lubitz SA, Sullivan LM, Sun JX, Levy D, Vasan RS, Magnani JW, Ellinor PT, Benjamin EJ, Wang TJ. Low serum magnesium and the development of atrial fibrillation in the community: the Framingham Heart Study. Circulation. 2013;127:33-8.
10. Molnar AO, Eddeen AB, Ducharme R, Garg AX, Harel Z, McCallum MK, Perl J, Wald R, Zimmerman D, Sood MM. Association of Proteinuria and Incident Atrial Fibrillation in Patients With Intact and Reduced Kidney Function. J Am Heart Assoc. 2017 Jul 6;6(7):e005685.
11. Dahlqvist S, Rosengren A, Gudbjörnsdottir S, Pivodic A, Wedel H, Kosiborod M, Svensson AM, Lind M. Risk of atrial fibrillation in people with type 1 diabetes compared with matched controls from the general population: a prospective case-control study. Lancet Diabetes Endocrinol. 2017;5:799-807.
12. Hu WS, Lin CL. Association between varicose vein and atrial fibrillation-a population-based study in Taiwan. Phlebology. 2022;37:535-539.
13. Rahimi M, Taban-Sadeghi M, Nikniaz L, Pashazadeh F. The relationship between preoperative serum vitamin D deficiency and postoperative atrial fibrillation: A systematic review and meta-analysis. J Cardiovasc Thorac Res. 2021;13:102-108.
14. Cha MJ, Seo HM, Choi EK, Lee JH, Han K, Lee SR, Lim WH, Park YM, Oh S. Increased Risk of Atrial Fibrillation in the Early Period after Herpes Zoster Infection: a Nationwide Population-based Case-control Study. J Korean Med Sci. 2018;33:e160.

Supplementary Table S2: Top 100 reasons for hospitalisation in atrial fibrillation patients, compared to controls, within 5 years pre incident AF diagnosis.


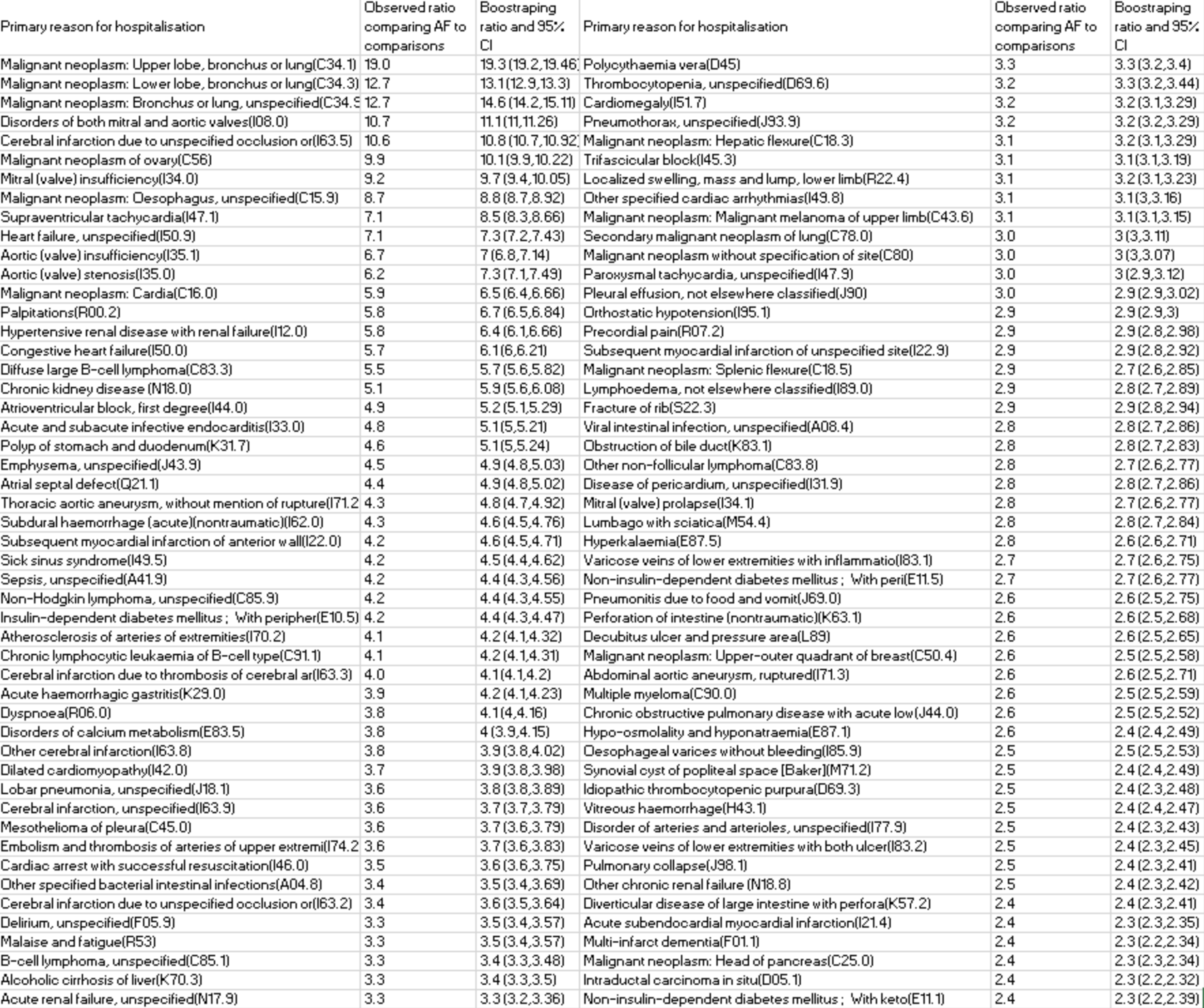


Supplementary Table S3: Top 100 reasons for hospitalisation in atrial fibrillation patients, compared to controls, in the 5 years post incident AF diagnosis.


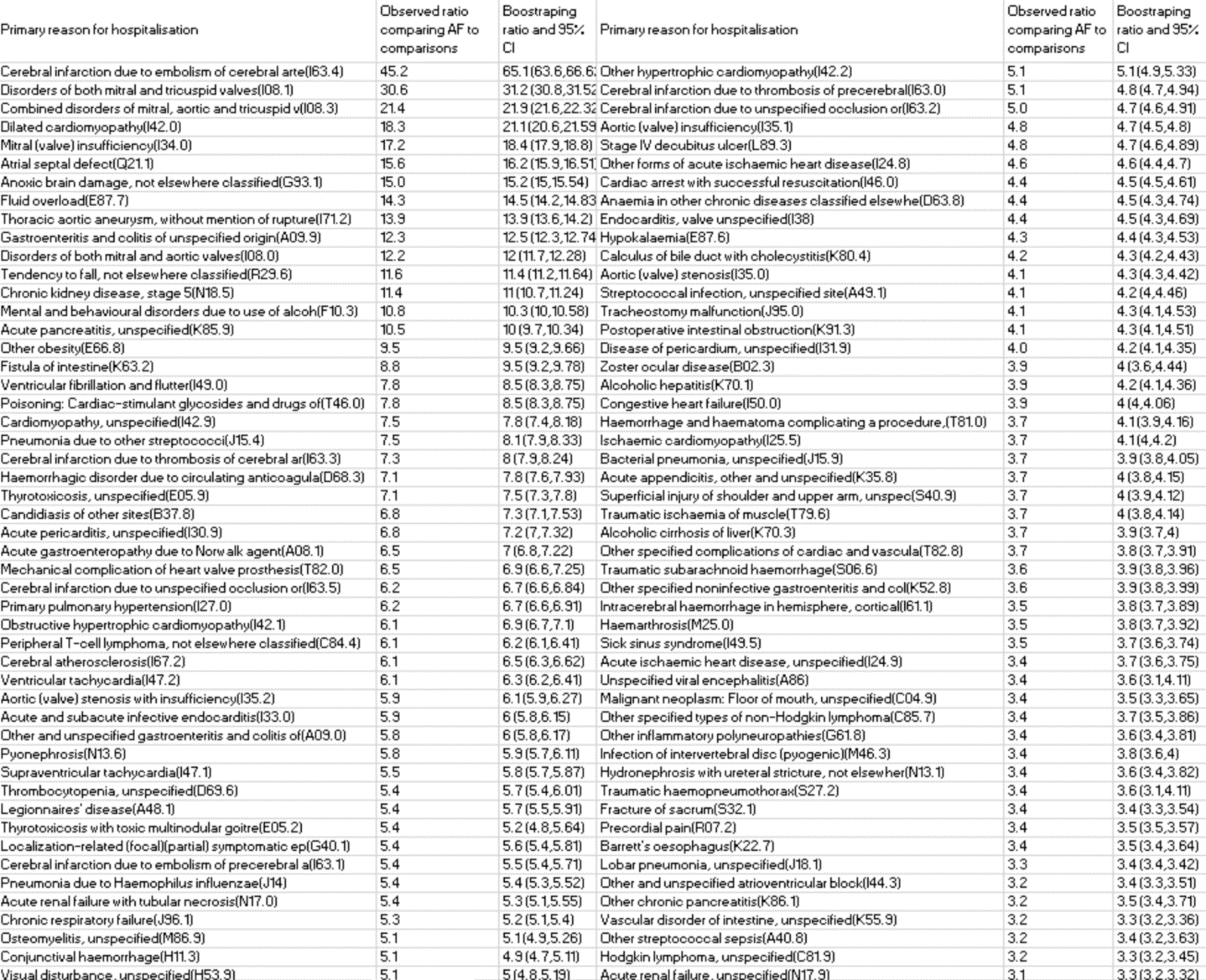


Supplementary Table S4: Top 100 reasons for GP consultations in atrial fibrillation patients, compared to controls, within 5 years pre incident AF diagnosis.


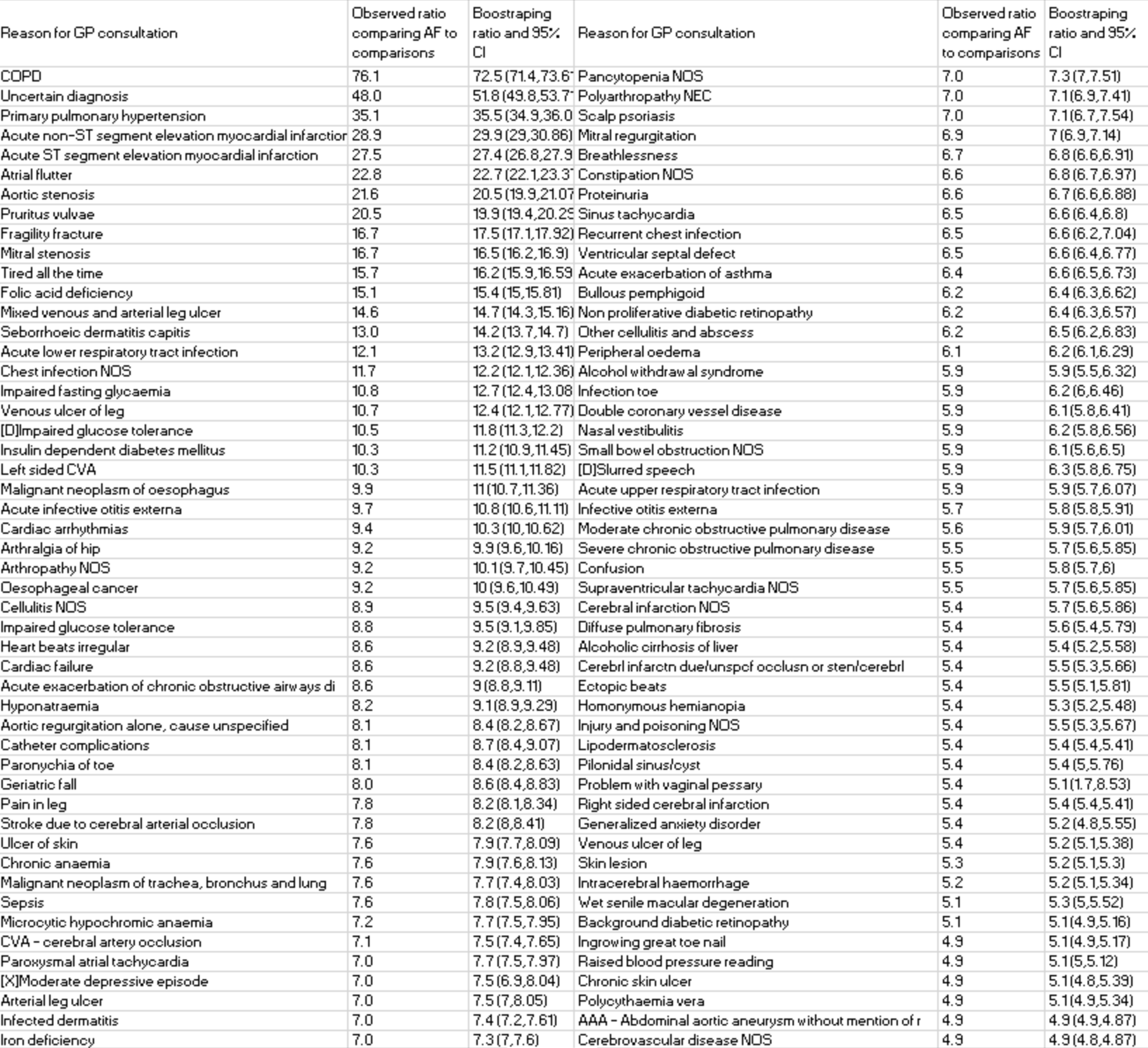


Supplementary Table S5: Top 100 reasons for GP consultations in atrial fibrillation patients, compared to controls, in the 5 years post incident AF diagnosis.


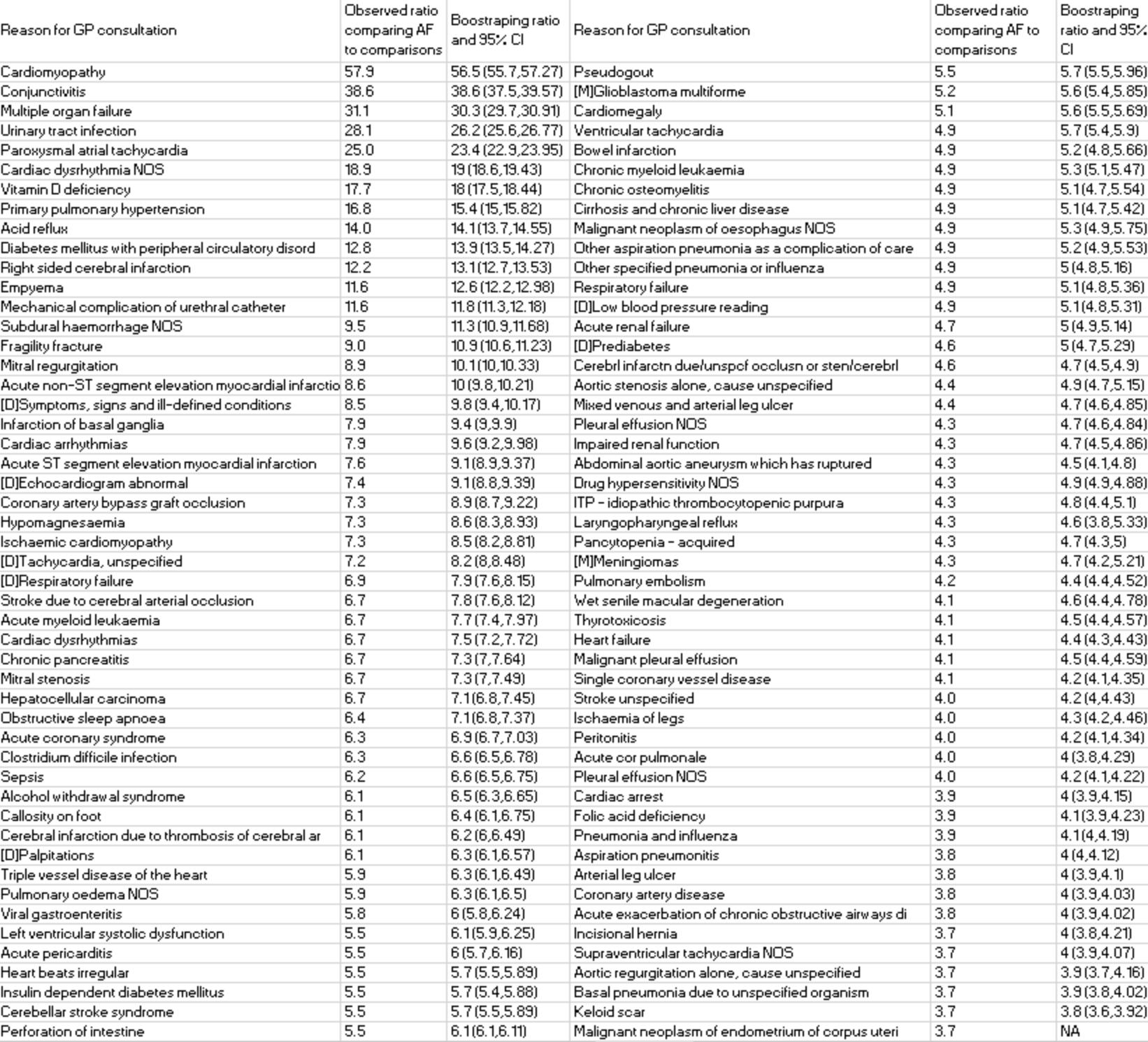

Supplement: Supplementary file 1 [file Table1.docx]
